# The Role of Machine Learning and Deep Learning Approaches for the Detection of Skin Cancer

**DOI:** 10.3390/healthcare11030415

**Published:** 2023-02-01

**Authors:** Tehseen Mazhar, Inayatul Haq, Allah Ditta, Syed Agha Hassnain Mohsan, Faisal Rehman, Imran Zafar, Jualang Azlan Gansau, Lucky Poh Wah Goh

**Affiliations:** 1 Department of Computer Science, Virtual University of Pakistan, Lahore 54000, Pakistan; 2 School of Information Engineering, Zhengzhou University, Zhengzhou 450001, China; 3 Department of Information Sciences, Division of Science and Technology, University of Education, Lahore 54000, Pakistan; 4 Optical Communications Laboratory, Ocean College, Zhejiang University, Zhoushan 316021, China; 5 Department of Statistics and Data Science, University of Mianwali, Mianwali 42200, Pakistan; 6 Department of Bioinformatics and Computational Biology, Virtual University of Pakistan, Lahore 57000, Pakistan; 7 Faculty of Science and Natural Resources, Universiti Malaysia Sabah, Jalan UMS, Kota Kinabalu 88400, Sabah, Malaysia

**Keywords:** classification, detection, deep learning, identification, machine learning, skin cancer

## Abstract

Machine learning (ML) can enhance a dermatologist’s work, from diagnosis to customized care. The development of ML algorithms in dermatology has been supported lately regarding links to digital data processing (e.g., electronic medical records, Image Archives, omics), quicker computing and cheaper data storage. This article describes the fundamentals of ML-based implementations, as well as future limits and concerns for the production of skin cancer detection and classification systems. We also explored five fields of dermatology using deep learning applications: (1) the classification of diseases by clinical photos, (2) der moto pathology visual classification of cancer, and (3) the measurement of skin diseases by smartphone applications and personal tracking systems. This analysis aims to provide dermatologists with a guide that helps demystify the basics of ML and its different applications to identify their possible challenges correctly. This paper surveyed studies on skin cancer detection using deep learning to assess the features and advantages of other techniques. Moreover, this paper also defined the basic requirements for creating a skin cancer detection application, which revolves around two main issues: the full segmentation image and the tracking of the lesion on the skin using deep learning. Most of the techniques found in this survey address these two problems. Some of the methods also categorize the type of cancer too.

## 1. Introduction

**Figure 1:**
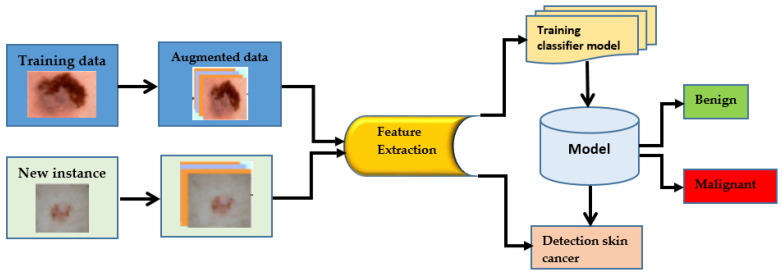
Process of cancer detection.

Skin cancer is a term used to describe a group of diseases in which abnormal skin cells grow uncontrolled and form tumors. These cancers are primarily brought on by unprotected skin damage to UV (ultraviolet) rays [1]. Melanomas make up just one percent of skin cancers. Skin cancers or basal cell carcinomas make up the remaining cases [2]. Due to its widespread occurrence and the potential for huge consequences, HPV is a significant health concern in the United States. In the United States, about five million different skin diseases are thought to be recorded annually. The rate of skin cancer has increased since the 1970s. Specialists use a variety of methods to find skin cancer [3]. To determine if a lesion is malignant, a trained physician will frequently use a predefined set of criteria, such as looking for concerning lesions, performing a dermoscopy, and then performing a biopsy. It could take some time before the person advances to the next level. Dermoscopy has improved the absolute accuracy of picture identification by fifty percent, from seventy-five to eighty-four percent. A doctor’s skills are also essential to making an appropriate diagnosis. For those who have skin conditions, manually diagnosing them is challenging and time-consuming [4]. Dermoscopy procedures can be analyzed using computer-assisted diagnosis when a certified expert is not accessible because of limited expertise. The differences between samples and researchers can be minimized with the help of automatic categorization. There were two major problems with the most effective computer-assisted skin-related picture categorization systems: insufficient data and the way the photos were taken. With today’s methodologies, significant preprocessing, segmentation, and feature extraction was necessary before skin image classification. AI-related changes are similar to those brought about by widespread technological adoption. Machine learning (ML) technology can be used for classification tasks rather than manually extracting features. You can save time and effort by doing this. More people are now trying to use ML methods to diagnose cancer, a recent trend. Machine learning algorithms have improved cancer prediction accuracy by 15% to 20% over the last two decades. Artificial intelligence’s deep learning discipline is fast growing and has many possible applications. One of the most powerful and widely used machine learning (ML) techniques for recognizing and categorizing pictures is deep learning, using specifically convolutional neural networks (CNNs), which are powered by state-of-the-art computational algorithms and massive datasets. Traditional ML techniques, which demand a lot of background knowledge and lengthy preparation stages, are no longer frequently used. Deep learning-based classifiers can identify skin cancer in images just as effectively as dermatologists. CNN’s can aid in developing computer-aided categorization systems for skin lesions used by dermatologists. There are not enough training sets for high-quality medical images, though. This is mostly due to a lack of photos of rare classes that are sufficiently annotated and described. Standard CNNs are less likely to work successfully with small data sets. There is a significant problem with processing costs for therapeutic applications because some researchers utilize exceptionally deep CNN models (Resnet152, for example, has 152 layers), which improve network classification performance. In addition, researchers use CNNs that have been trained to categorize skin lesions. By doing this, data overfitting is avoided. Features obtained from real-world photo datasets are added to trained convolutional neural networks (CNNs) (such as ImageNet). Additionally, CAD makes identifying and treating tumor diseases easier and more cost-effective. Imaging methods such as MRI, PET, and X-rays are often used to diagnose organ problems. Skin lesions were initially identified visually; CT, dermoscopy image processing, clinical screening, and other methods allowed this. Less experienced dermatologists are more likely to misdiagnose skin lesions. The procedures used by doctors to review and understand images of lesions take a lot of time and are often subjective and inaccurate. This is mostly because it is challenging to describe skin problems on camera fully. Knowing precisely where pixels are situated within skin lesions is crucial to analyzing images and diagnosing problems with lesions [5]. The ability of CADI and CPS systems to identify skin cancer has considerably increased with the integration of machine-learning approaches to computer vision. Image preprocessing and lesion image categorization are two of the most important elements in cancer detection and diagnosis. However, the outcome and mortality rates of skin cancer must be improved through early detection. For diagnostic tests or assessments of patient response to therapy (such as for cancer), this approach is unsuitable. To enhance and speed up the diagnostic decision-making process, many doctors are using AI in medicine [6]. Most artificial intelligence research for clinical diagnosis has either ignored or inadequately addressed the necessity for accurate assessment and reporting of future problems, even though there have been encouraging signals of development in this field. Numerous diseases can be recognized quickly, precisely, and consistently using computer-aided design [7]. Additionally, CAD makes identifying and treating tumor diseases easier and more cost-effective. Imaging methods such as MRI, PET, and X-rays are often used to diagnose organ problems. In the past, methods such as clinical screening, computed tomography (CT), dermatoscopy image processing, and others were used to detect skin lesions. Dermatologists with less experience are less proficient in correctly diagnosing skin lesions. The procedures used by doctors to interpret and analyze images of lesions take a lot of time and are often subjective and inaccurate. This is mostly because it is challenging to depict skin imperfections on camera accurately. One approach to a skin cancer diagnosis is shown in Figure 1.

This study of dermatology and medicine at large are the basis of a variety of knowledge that can revolutionize customized healthcare, such as the availability of clinical records, patient population data, and imagery testing and evaluation data [1,2]. The fields of study frequently related to the shortened term omics are now prevalent in dermatological translation research using evidence from genomes, epigones, transcriptomes, proteomes and microbiomes [3]. Recent developments in faster computing and cheaper storage have facilitated the creation of human-like intelligent machine learning (ML) algorithms for various dermatology apps. Dermatologists must have a basic understanding of artificial intelligence and ML to test the usefulness of these new technologies. Skin cancer, with over five million cases diagnosed annually, is the most prevalent cancer in the U.S [1]. With over 100,000 new possibilities in the United States and about 9000 deaths yearly, basal cell carcinoma is the deadliest type of skin disease [2]. U.S. health care costs more than USD 8 billion [4]. Skin cancer represents a massive danger to public health worldwide as well. Over 13,000 new cases of melanoma arise annually in Australia, resulting in over 1200 deaths [5]. More than 20,000 deaths yearly are triggered by melanoma in Europe [6]. Early diagnosis is essential to combat the elevated mortality of melanoma. Highly trained dermoscopic clinicians are required to diagnose melanoma reliably and early, but the number of experts has not met the need [7]. A Dermot scope is a sophisticated form of high-resolution skin imagery that decreases the reflectivity of the skin surface to enable clinicians to see deeper underlying structures. This system displays diagnostic precision of 75% to 84% by specially qualified clinicians [8]. However, the identification efficiency declines dramatically when the physicians are not well qualified.

## 2. Literature Review

The use of technology in early cancer diagnosis has shown that it is possible to get around the limitations of the manual process, opening up a new field of study. To better understand the reader’s understanding of the topic at issue and the current state of knowledge, this section summarizes various pertinent studies. Deep learning techniques have outperformed traditional machine learning techniques in many situations. Over the past couple of decades, deep learning has fundamentally changed how machine learning works. The topic’s novel feature is using artificial neural networks in machine learning. This approach was based on how the brain works and is organized [9]. Researchers looked at the data to see how accurate computerized methods are [10]. Researchers revealed a more accurate way to identify melanoma skin cancer [11]. A non-linear segmentation insertion surface was used to generate fake images of melanoma. Data augmentation was used to create a new collection of skin melanoma datasets using ceroscopy photos from the publicly accessible PH2 dataset. The Squeeze Net deep learning model was trained using these photos. The study showed a major improvement in melanoma detection. The VGG-Seg Net technique was suggested to extract a skin melanoma (SM) region from a dermatoscopy image [12]. Important performance metrics were developed by comparing the segmented SM to reality. The suggested method was reviewed and verified using the industry-recognized ISIC2016 database. The Classification of skin cancer was determined by researchers using both machine and human intelligence. The 300 skin lesions confirmed by biopsy were categorized into five kinds by a CNN expert and 112 German dermatologists. The two separate sets of diagnostics were integrated into a single classifier using boost. The results revealed that 82.95%of the time, humans and machines were right about more than one class. Deep learning-based technology can identify benign and malignant tumors [13]. The method was tested using HAM10000 images from the 2018 ISIC, 2019 ISIC, and 2020 ISIC in a typical laboratory setting. The InSiNet framework outperforms the other methods using the ISIC 2018 dataset, with accuracy rates of 94.59%, 91.89%, and 90.549%, respectively. Researchers created a deep learning method to identify early-stage melanoma that uses a region-based convolutional neural network and fuzzy k-means clustering [14]. The effectiveness of the suggested method in assisting dermatologists in the early recognition of this potentially lethal condition was assessed using a variety of clinical pictures. The effectiveness of the proposed method was assessed using the ISIC-2017, PH2, and ISBI-2016 datasets. It was more accurate on average than the top methods at the time (95.40%, 93.11%, and 95.60%). Convolutional neural networks are a type of deep learning model that has been discovered to be more effective than conventional techniques for detecting images and features [15]. They have also been used in the medical field, where they have helped patients overcome incredible obstacles and perform amazing accomplishments. There are currently many DL-based medical imaging systems that clinicians and specialists can use to identify, treat and track the progress of cancer patients. To distinguish between melanoma and non-melanoma skin lesions, we developed the Lesion classifier. The system’s efficacy was evaluated using the ISBI2017 and PH2 skin lesion databases. Tests on the ISIC 2017 and PH2 datasets showed that the proposed approach was 95% accurate. To determine whether 100 skin lesions were benign or malignant, researchers created a set of deep learning algorithms using data from the International Skin Imaging Collaboration (ISIC)-2016 dataset [16]. Overall, they were more accurate than specialists. Compared to specialists, who had an accuracy rate of 70.5% and a specificity rate of 59%, they had a 76% accuracy rate and a 62% specificity rate. A dermoscopic dataset of over 100,000 benign lesions and melanoma images was used to train the InceptionV4 deep learning algorithm. Then, the results were contrasted with those of 58 dermatologists. There were two groups, depending on the diagnosis level [17]. Dermoscopy was the only technique used in the first level, but it was combined with patient clinical data and images in the second level. Dermatologists’ reports indicate that the first level’s median sensitivity was 86.6%, and its median specificity was 71.3%. Sensitivity and specificity climbed to 88.9% and 75.7%, respectively, at level II. There was a statistically significant rise in specificity (*p* 0.05). The increase in sensitivity, however, was not statistically significant, according to statistical analysis (*p* = 0.19). The CNN with deep learning produced a much more precise receiver operating characteristic curve than dermatologists at level (*p* 0.01). CNN performed better than the majority of dermatologists in this study. This suggests that CNN could be used to help with melanoma detection using dermoscopy images. In this study (MClass-D), 157 dermatologists’ performances on 100 dermoscopic images was compared using a convolutional neural network (ResNet50) (MClass-D). Dermatologists’ sensitivity and specificity were lower than those of deep learning, which had a sensitivity of 84.2% and a specificity of 69.2%. In a head-to-head comparison, CNN outperformed 86.6% of the dermatologists in the study. Each subgroup of dermatologists performed better depending on their experience in categorizing dermoscopy images of melanoma. Thus, CNN might be able to help dermatologists make an accurate melanoma diagnosis. Deep learning algorithms exceeded human groups with beginner and intermediate raters. The trained combined CNN had a significantly larger area under the ROC curve than dermatologists. Compared to general dermatologists, it correctly identified more cases, but identified fewer than experts with more than ten years of experience [18]. The sensitivity and specificity of the ResNet50 deep learning system’s capability to categorize skin lesions into specific groups were assessed and 112 German dermatologists. Dermatologists had a sensitivity of 74.4% and a specificity of 59.0% for correctly classifying skin lesions. The algorithm was accurate 91.3% of the time at the same sensitivity level. Dermatologists correctly classified an image into one of the five diagnostic categories with a sensitivity of 56.5% and a specificity of 89.0%. The algorithm offered 98.8% specificity at the same level of sensitivity. The deep learning algorithm generally performed better on the main outcome than dermatologists (*p* 0.001). The secondary outcome comparison revealed that, except for basal cell carcinoma, the algorithm consistently outperformed dermatologists [19]. Dermatologists helped test an InceptionV4-based deep learning architecture using 100 dermoscopy images. Dermoscopic images made up Level I, while clinical details, a dermoscopic image, and a clinical close-up image made up Level II. The deep learning system’s sensitivity and specificity were both 95% when compared to Level I dermatologists. Dermatologists’ average specificity remained constant while their average sensitivity increased to 94.1% with Level II information [20]. An online open study was used to ascertain what was wrong with the dermatoscopy photographs. The researchers evaluated the average performance of 139 AI algorithms against 511 human readers on a set of 1511 images from the ISIC 2018 competition. We contrasted human and machine learning algorithm diagnoses, each of which fit into one of seven predefined categories. We evaluated the effectiveness of various diagnostic techniques. The participants included 55.4% board-certified dermatologists, 23.1% dermatology residents, and 16.2% family physicians. A human and an algorithm performed diagnostic tasks differently on average by 2.01 points, and there was a statistically significant difference (*p* 0.0001). This suggested that AI algorithms could diagnose illnesses more precisely than people [21].

## 3. Methodology

**Figure 2:**
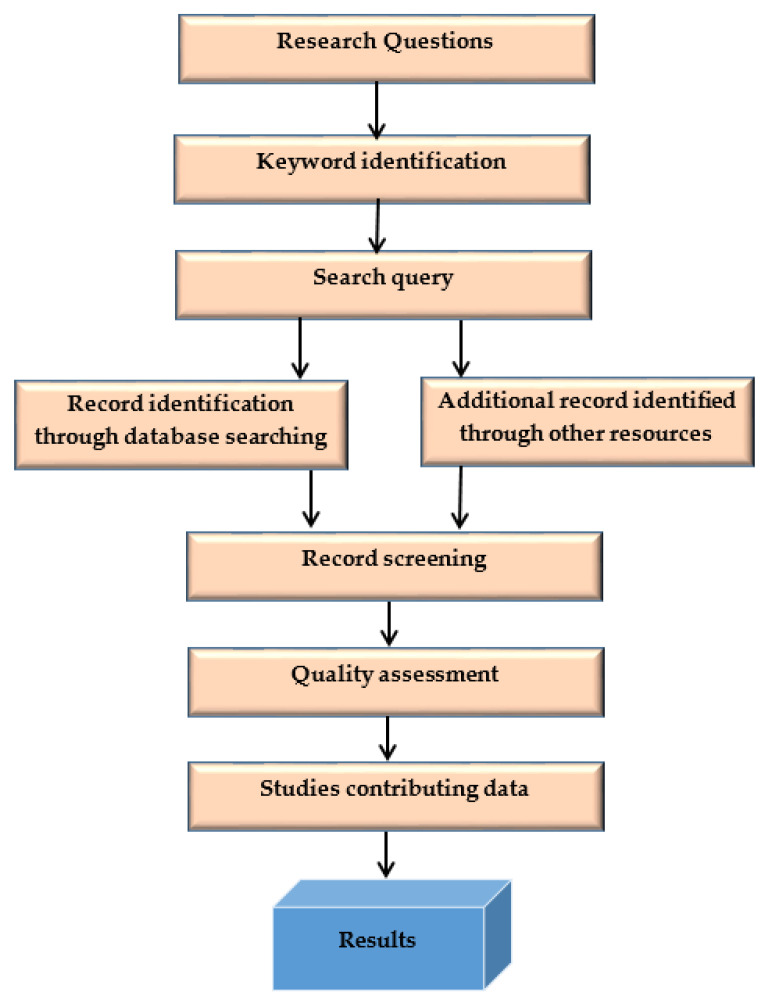
SLR methodology process.

This systematic literature search (SLR) methodology process is shown in Figure 2.

### 3.1. Problem Statement

The primary problem is to outline the pros and cons of the method used to identify and classify skin cancer disease from the medical images and compare them for their efficiency.

### 3.2. Research Question

The research questions are listed in Table 1.

Kitchenham and Charters [8] defined guidelines for computer engineering systematic literature reviews, which this study follows. A systematic literature review uses a disciplined and repeatable technique to give a reliable and objective analysis of a research topic. The standards used are high-level and do not consider how the study issue affects the evaluation process. The review strategy is depicted in Figure 1. It is a different set of functions that aid in managing the review process; the author defined the protocol. In the following sections, we describe each step we summarized in Figure 1. To identify proper, efficient deep-learning skin cancer classification methods, we conducted a large-scale survey of studies of machine learning methods. This survey aims to understand different algorithms’ specific requirements and performance characteristics. We compared different categories of algorithms, referred to as machine learning and deep learning methods. The following questions are formulated to carry this SLR.

### 3.3. Keyword Identification

Primary and secondary keywords are presented in Table 2.

### 3.4. Search Query

Keywords are important words used to describe the subject and field of study in the titles and abstracts of research articles. The literature is searched using keywords to obtain solutions to the study questions. The literature is retrieved from the database and other sources using these keywords. There is a list of the keywords that were used to discover the primary studies for this SLR in the “Abstract” section. Finding out what keywords are most commonly used is the first step in developing a search strategy. To locate the primary keywords, the search begins by looking at the papers of well-known scholars. These keywords, as well as phrases that are similar to them, make up search queries. The research process is made more accessible by using the provided keywords. If you use the appropriate terms, your research will move much faster. The search query process is presented in Table 3.

#### 3.4.1. Year-Wise Papers Selection

The final papers selected from 2018 to 2022 are presented in Table 4.

**Figure 3:**
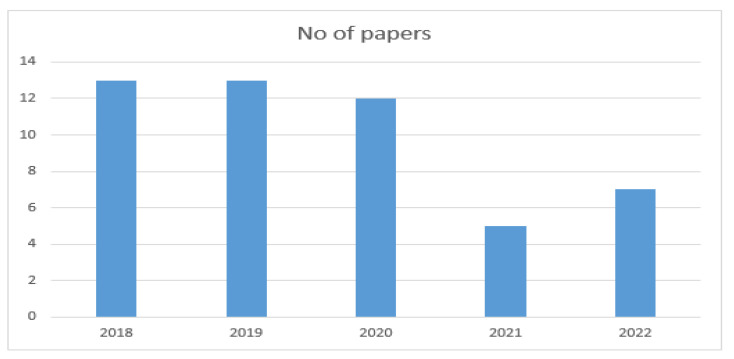
Year-wise selection of papers.

The year-wise selection of papers is shown in Figure 3.

#### 3.4.2. Final Paper Selection

**Figure 4:**
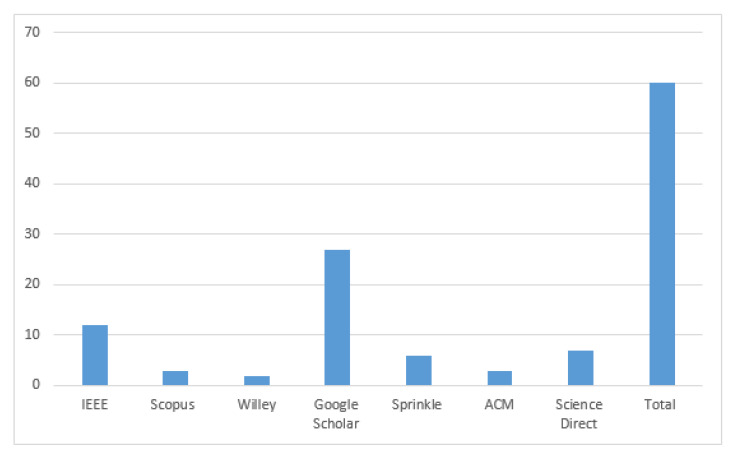
Final paper selection.

The selection of final papers is presented in Table 5 and Figure 4.

### 3.5. Inclusion/Exclusion Criteria and Quality Assessment

The following steps are followed for the quality assessment of papers:

- Initially, an extensive list of papers is obtained following the keyboard search.
- Publisher-based filtering is performed. Only journal/workshop papers are included
- Only papers from renowned publishers are included, such as springer, IEEE, A.C.M., Wiley, etc.
- Papers from Indian journals are excluded.
- Title-based filtering is performed, and the rest of the papers with the keywords in their titles are excluded.
- Then abstracts of the papers are read, and those papers are included, which describe comparing deep learning methods’ performance in classifying skin cancer.
- After that, papers are searched for answers to our questions. Articles are included if the solution to any question is found; otherwise, it is excluded.

The following factors are taken into account when sorting the papers:

- If the title contains at least one keyword, the mark is 1; otherwise, it is 0.
- If the abstract defines a performance evaluation metric, the mark is 1; otherwise, the mark is 0.
- If the introduction and conclusion discuss performance measurements, the mark is 1; otherwise, the grade is 0.
- If the work describes a comparison with at least one earlier study, the mark is 1; otherwise, it is 0.
- In the end, papers with a score >=3 are included in the results.
- Workshop indicates A, conference indicates B, journal indicates C,
- The quality assessment of the papers is presented in Table 6.

Cancer develops when tissues in a particular organ or body part grow out of control. One of the cancers with the fastest global spread is skin cancer.

Skin cancer can develop due to the uncontrolled and rapid spread of unwanted skin cells. Potential treatments can be successful only if cancer is identified early and diagnosed correctly. Most deaths from skin cancer in developed countries are caused by melanoma, the fatal type of disease. Deep learning significantly outperforms human experts in several computer vision competitions at early skin cancer detection, resulting in lower mortality rates. It is possible to combine powerful formulations with deep learning methods to achieve excellent processing and classification precision. The deep hybrid learning (DL) model for classification and prediction is used to identify early cancer indicators in lesion images. Preprocessing and classification are essential in the system under consideration. The overall image intensity is increased during the preprocessing stage to reduce the counterpoint between shots. The image is normalized and scaled during this process to match the size of the training model. The proposed model’s performance was assessed in the comparative studies using several different metrics. As quantitative measures, precision, recall, F1, and the area under the curve were used (AUC).

Skin cancer has increased in occurrence over the past ten years. Since the skin is the body’s largest organ, skin cancer is probably the most common in people. Only when skin cancer is found early can it be effectively treated. The earliest signs of skin cancer can now be found more easily than ever, thanks to computational methods. According to one report, there are several non-invasive ways to look at skin cancer symptoms. The various shortcomings of methods for dividing and classifying skin lesions were examined. An improved method for melanoma skin cancer diagnosis is described in clause [39]. An implantation manifold with non-linear embedding was used to create synthetic images of melanoma. Using the data augmentation strategy, a new set of skin melanoma datasets were created using dermoscopic scans from the public PH2 dataset. The Squeeze Net deep learning model was trained using the improved images. Experiments showed a significant improvement in melanoma detection accuracy (92.18). A skin melanoma (SM) region could be extracted from a digital dermatoscopy image using the VGG Sag Net algorithm.

Comparing the segmented SM to the actual data revealed critical performance parameters (GT). The proposed method was tested, and its accuracy was verified using the industry-recognized ISIC2016 database. The use of machines to assist in early cancer detection has shown the shortcomings of the manual method and given rise to a new area of study. This section summarizes a few pertinent studies to give readers a sense of the subject at hand and an idea of the current situation. In many instances, deep learning techniques have outperformed more traditional machine learning methods. Over the past several decades, deep learning has significantly changed the field of machine learning. Machine learning’s most cutting-edge technology is artificial neural networks. This approach was based on how the human brain works and is organized. In several fields, CNNs and other deep learning models have been shown to outperform traditional approaches. Examples include the ability to recognize features and images. They have achieved outstanding results and excelled under pressure in the medical field.

A wide range of DL-based medical imaging systems are now available to medical professionals, which can help with cancer diagnosis, treatment planning and treatment efficacy assessment. With the help of CAD (Computer-aided design) software, diagnosing various diseases can be accelerated, uniformed and made more precise. Additionally, CAD makes detecting and preventing cancer diseases simpler and more affordable. Imaging methods such as magnetic resonance imaging (MRI), positron emission tomography (PET) and X-rays are used to examine organ issues in people. In the past, clinical screening, image analysis with computed tomography (CT), dermatoscopy and other techniques could be used to find and diagnose skin lesions. The difficulty in diagnosing and reporting NMSC, on the other hand, may cause the second number to be significantly lower than the actual number. Numerous factors contribute to the high death rate from skin cancer, including late diagnosis brought on by ambiguous symptoms, ineffective screening techniques, a lack of sensitive and specific biomarkers for early diagnosis, prognosis, and treatment monitoring, as well as ignorance of the mechanisms by which these tumors develop drug resistance. As a result, the COVID-19 pandemic has taken center stage in daily clinical practice. Due to the difficulty in treating those with CM and other skin cancers and the delay in diagnosis, there has been an increase in the rates of illness and death and an increase in the financial burden on the healthcare system. Future cancer treatments may benefit from personalized medicine, which determines the best course of action for each cancer patient based on their particular molecular characteristics. The personalized approach determines the likelihood that the tumor will spread and the best course of treatment using a multidimensional biochemical analysis of several biological endpoints. Droplet digital polymerase chain reaction (DDPCR) has recently become a popular omics technology due to its ability to identify and measure minuscule amounts of nucleic acids in various biological samples. This is crucial for subtyping cancer, predicting outcomes, and keeping track of the disease’s progression.

The fact that skin cancer is frequently detected too late is a major contributor to its high mortality rate. There are not enough trustworthy screening techniques, as well as not enough sensitive and precise biomarkers for early diagnosis and prediction of the course of these tumors, and it is unclear how drugs lose their effectiveness. Nearly all thin lesions in Australia that were surgically removed after five years are still alive (depending on the presence of ulceration). As a result, survival rates drastically decrease as tumor thickness increases. Only 54% of people with CM tumors thicker than 4 mm will survive. The health of the lymph nodes in the affected area can predict future outcomes even in the earliest stages of melanoma. Unnoticeable lymph node metastases can be found using a sentinel lymph node (SLN) biopsy. This makes it possible to remove lymph nodes early to stop the cancer’s spread. The identification of biomarkers that can be used in the clinical management of oral cancer, ovarian cancer, and esophagogastric cancer, however, has been made possible by the promising outcomes of DDPCR assays for circulating miRNAs in other categories of tumors. There is a great deal of optimism that more efficient and individualized treatments will be found for these patients when DDPCR techniques are used in biomedical and translational research on skin cancer.

## 4. Results

### 4.1. RQ1: What Are the Features and Advantages of Recently Developed DL Methods for Skin Cancer Classification?

Initially, most articles conducted two operations on the skin lesion and graded it into melanoma and the initial stage. Most studies analyzed in this report centered on an evaluation based on differential disease classification. Upon diagnosing cancer such as melanoma, a suitable type was further tested. Melanoma comprises four significant forms, nodal melanoma, acral lentiginous, and lentigo malignant, which are superficially propagated. Deep learning methods to detect a specific type were trained on lesion images. All forms, positions and lesion objects concerning proportions and colors exist. Superficial melanoma has an obscure hue on the skin with an unusual line.

In comparison, both in scale and color, lentigo malignancy and lentigo entities have been formed irregularly. If the disease has not been described as benign, the disease is graded as dermal, melanocytic or epidermal in three distinct forms. Such forms fall into the heading of a non-cancerous skin condition similar to melanoma. Moreover, the most used technique is DCNN, followed by Fr CN and transfer learning, which is almost reported for every data set. A comparison of different DL methods is presented in Table 7.

### 4.2. RQ2: What Data Sets Are Used in Skin Cancer Detection Methods Evaluations?

To detect skin cancer, several computer-based methods have been suggested. A strong and reliable collection of dataset images is necessary for assessing diagnostic performance and assuring expected outcomes. Images of nevi and melanoma lesions are the only images in skin cancer databases currently available. Insufficient data types and small datasets make it challenging to train artificial neural networks to classify skin lesions. Although patients frequently have a wide range of non-melanocytic lesions, previous research on automated skin cancer diagnosis has mainly focused on melanocytic lesions.

The list of the datasets used for skin cancer detection is explained in Table 8.

Other datasets are not publicly and freely available, which is why they are considered private and not included in this SLR.

### 4.3. RQ3: What Are Future Challenges Reported in This Domain?

#### 4.3.1. Extensive Training

One of the most important problems is that skin cancer detection using neural networks is not as efficient as it could be. The system must undergo extensive training, which requires a lot of time and very powerful hardware, before accurately assessing and interpreting the features from image data.

#### 4.3.2. Variation in Lesion Sizes

Another issue is that lesions come in different sizes. Many images of benign and malignant melanoma lesions were taken in the 1990s by an Italian and Austrian research team [55]. Doctors who attempted to locate lesions were accurate between 95% and 96% of the time. However, initially, the diagnostic procedure was more challenging and faulty when the lesions were only 1 mm or 2 mm in size [56].

#### 4.3.3. Images of Light-Skinned People in Standard Datasets

Caucasians, Europeans, and fair-skinned Australians are represented in the standard dataset. To more accurately identify skin cancer in people of color, a neural network can be trained to consider skin color [14]. This is only feasible, though, if enough images of people of color are included in the neural network’s training data. Lesion images from enough people with dark and light skin are needed to improve the precision of skin cancer detection systems [57].

#### 4.3.4. Small Interclass Variation in Images of Skin Cancer

Medical images are essentially identical to other kinds of images. The differences between cats and dogs are significantly less when compared to images of melanoma and non-melanoma skin cancer lesions [58]. It might be challenging to tell a pimple from a specific skin cancer called melanoma. Some diseases’ lesions are hardly distinguishable from one another. Due to a lack of differences, image analysis and classification are incredibly difficult [59].

#### 4.3.5. Use of Various Optimization Techniques

In order to do preprocessing and automatically detect skin cancer, the boundaries of the lesion must be identified. Automated skin cancer diagnosis systems should perform better when using optimization techniques, such the artificial bee colony algorithm, ant colony optimization, social spider optimization, and particle swarm optimization.

#### 4.3.6. Unbalanced Skin Cancer Datasets

Real-world data are highly biased, which makes identifying skin cancer challenging. The number of images for each type of skin cancer differs widely when data sets are not symmetrical. It is challenging to conclude skin cancer from dermoscopy images because there are many images of common types but few images of rare types [60].

#### 4.3.7. Lack of Availability of Powerful Hardware

The Neural network needs a lot of hardware resources and a strong graphics processing unit to extract specific areas of an image of a lesion, which is required for a more precise skin cancer diagnosis. Deep learning is difficult to train for skin cancer detection due to low processing power.

#### 4.3.8. Lack of Availability of Age-Wise Division of Images in Standard Datasets

Merkel cell carcinoma, basal cell carcinoma, and squamous cell carcinoma are common after age 65. The dermoscopy databases that are currently available follow industry standards and contain pictures of children. However, until neural networks have seen enough images of people over 50, they will not be able to diagnose skin cancer in people over 50 correctly [61].

#### 4.3.9. Analysis of Genetic and Environmental Factors

This type of skin cancer is genetically more likely to affect people with fair skin, light eyes, red hair, many moles, and a family history of melanoma. The likelihood of developing skin cancer dramatically rises when these environmental risk factors, such as extended exposure to UV light, are combined with genetic risk factors. Deep learning methods can be enhanced by including these elements [62].

Different studies have identified future challenges in this domain, as shown in Table 9.

### 4.4. RQ4: What Are the Machine Learning and Deep Learning Approaches for Skin Cancer Detection?

**Figure 5:**
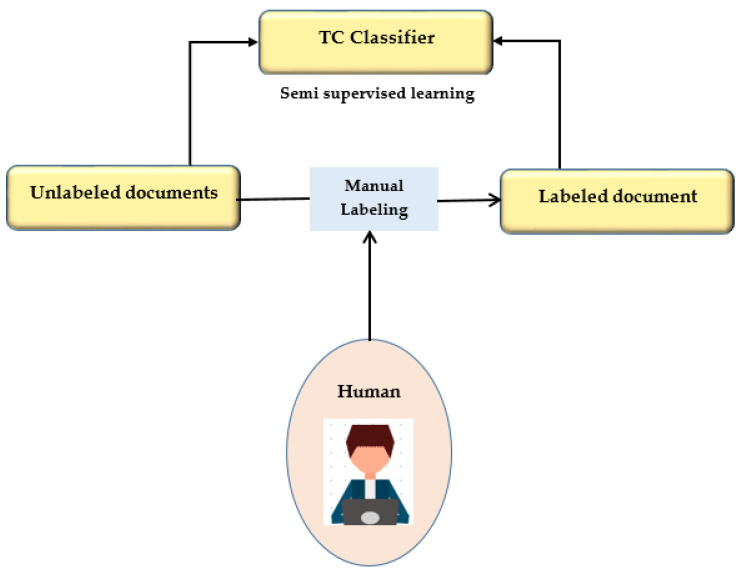
Semi-supervised learning.

This study presents a semi-supervised learning technique using two iterations of preprocessing and segmentation to separate lesions from dermoscopy images autonomously. Non-linear regression and the CLACHE algorithm are used to fix the image’s unequal lighting during the preprocessing stage of picture scaling [52]. by classifying pixels according to how their RGB color space appears, k-means clustering is used to increase the lesion prediction’s accuracy. A unique strategy is suggested for finding lesions that combine deep learning and local description encoding. A wide range of feature values that can be applied to different lesions can be quickly generated using this model. The publicly available ISBI 2016 dataset was used to assess the proposed model [32]. Semi-supervised learning is presented in Figure 5.

**Figure 6:**
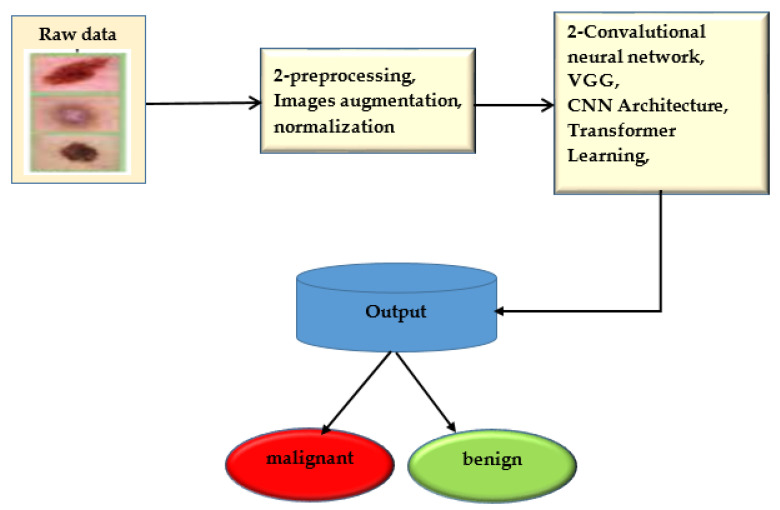
Convolutional neural network.

The convolution neural network was trained to use deep learning to anticipate minor skin changes using dermoscopy images. An initial dermoscopy screening is frequently used to diagnose skin cancer. A biopsy and histopathological analysis are then completed. The proposed framework correctly categorizes lesions using a novel regularized binary classifier [39]. To assess performance, the area under the curve for nevus images is calculated and contrasted with the area under the curve for lesion images. The convolutional neural network is presented in Figure 6.

The effectiveness of the suggested approach depends on the user’s knowledge and skill level. This project aims to make manual analysis less confusing and unpredictable. Researchers created a deep learning model based on lesion patterns for automatic melanoma recognition and lesion segmentation from images of skin lesions, combining a variety of hypotheses into one evaluation using a variety of deep learning algorithms [53]. It has issues because, before alerting a patient, a single doctor would frequently seek the advice of other specialists to pass and confirm the accuracy of the diagnosis. Numerous deep learning techniques were created using the same dataset and a significant amount of data improvement. Inception-v4, ResNet-152, and DenseNet-161 are only a few of the convolutional neural networks trained to recognize the difference between melanoma and seborrheic keratosis images. U-Net and U-Net with VGG-16 Encoder were used to construct segmentation masks for the lesion, both of which required training.

The CNN model is used as a kind of identification approach in dermoscopy images from the HAM10000 dataset to identify skin cancer. The results showed that the CNN model could achieve excellent accuracy when this dataset was used to train and test it. The authors of a different study assessed the effectiveness of various machine learning (ML) algorithms for breast cancer diagnosis using data from the UCI machine learning repository [54]. To choose which features to use, they used information-gain and relief. They then entered these features into algorithms including SVM, RF, RNN and CNN, to improve classification accuracy. The outcomes showed that RNN and other deep learning algorithms are more effective in identifying cancer than earlier methods.

As techniques that can be used independently to improve classification effectiveness and boost the precision of melanoma detection, a CNN is used to identify the different aspects of the lesion, and a deep learning technique based on the U-Net algorithm is used to separate the lesion area from dermoscopy images. Melanomas are categorized, and the malignancy of the tumor is determined using the VGG16 Net method. Both groups, finding segmented images and non-segmented images, are given. The ISIC 2016 data set found that deep learning-based categorization works more accurately when segmented pictures are used [55]. Deep learning-based techniques such as neural networks and feature-based algorithms can be used to find skin lesions more accurately by integrating probabilistic graphical models into this network [56]. PH2 and ISIC 2019 data should be used to train the network. The procedure is finished after NN architecture analysis and examination of training metrics such as accuracy, specificity, sensitivity, and the Dice coefficient. Using transfer learning based on CNN’s design, they can identify the different types of lesions. To identify what is in a photo, the proposed system employs a traditional technique that extracts a few elements from the image before classifying it. The categorizing procedure makes use of support vector machines. Due to the high incidence of skin cancer in western countries, especially the United States, the proposed technique is essential. There are 12 million people with cancer, and the suggested method provides more precise results. In the United States, an additional million cases of skin cancer are anticipated to be diagnosed by 2020. A dermatologist’s skilled eye is the best way to identify malignant cells and skin cancer. Because of this, it is challenging to provide everyone with high-quality dermatological care, and many people wait until their disease has grown worse before seeking it. A biopsy is a common procedure for making a variety of diagnoses. A little skin is removed during a biopsy and submitted to the lab for analysis. This is time-consuming and usually highly frustrating. Screenings with computer assistance can now find skin cancer in its earliest stages. Ordinary digital cameras and video recorders can capture images of tiny objects. The following classification of these images as measurable ones, commonly used in computer processing, is presented here. Poor lighting and artefacts, including skin lines, highlights, repetitions and hair, are potential problems in medical photography. Researching skin lesions is exceedingly challenging because of these barriers. On a computational level, preprocessing, trend detection, character selection, feature extraction and skin cancer detection are all conceivable. Malignant melanoma, the worst type of skin cancer, is on the rise. Due to artefacts, low contrast and the fact that skin cancer resembles a mole, scar, etc., it may be challenging to identify it from a skin lesion. Hence, lesion detection systems that are accurate, efficient and effective are used for automated skin lesion identification [57]. Early skin lesion detection is possible with the help of the ABCD rule, GLCM, and HOG feature extraction. Preprocessing improves the clarity and quality of skin lesions and eliminates abnormalities such as skin and hair color. Different parts of the lesion were made GAC, and each can be used separately for feature extraction. The ABCD scoring system was used to determine the features of symmetry, border, color and diameter. The texture of the object was determined using the HOG and GLCM programmed. Classifiers use characteristics to assess whether a skin lesion is benign or cancerous. They then use machine learning methods, such as support vector machines, k-nearest neighbors, and naive Bayes classifiers. The International Skin Imaging Collaboration (ISIC) scanned 328 benign and 672 malignant skin lesions for this study. The results showed an AUC of 0.94 and a classification accuracy of 97.8% using SVM classifiers. The test’s accuracy rate was 86.2%, with 85% confidence in its results [55]. Identifying benign from malignant tumors is the main goal of skin cancer research. Melanoma subtypes, on the other hand, have not previously drawn much attention. Dermoscopy and deep learning were used in this study to see if they could help detect AM and other types of melanoma. In this study, deep learning was used to learn how to recognize skin cancer. Using a collection of dermoscopy photos, we classified skin lesions. Several innovative image processing and information-adding methods have made AM identification easier. We used to seven-layer deep convolutional networks to build our model. We assessed the performance of our model using transfer learning on two different datasets, Alex Net and ResNet-18. With our new model, we could produce findings that were more accurate than 90% of the time for benign nevus and AM. We also avoided a 97% reduction in approach size because of transfer learning. Based on our research, we concluded that our skin cancer classification system was reliable and accurate. According to our research, dermatologists could use the suggested method to identify AML, which would be essential for patient therapy. Different machine and deep learning approaches and their results are shown in Table 10.

## 5. Findings

This systematic review discusses various deep learning algorithms for skin cancer detection and classification. These methods are all non-invasive. Preprocessing and picture segmentation are followed by feature extraction and classification to detect skin cancer. For the classification of lesion images, this review focused on ANNs, CNNs, KNNs and RBFNs. Each algorithm has its own set of benefits and drawbacks. Choosing the right classification technique is the most important factor in achieving the best results. However, when it comes to identifying picture data, CNN outperforms other types of neural networks since it is more closely tied to computer vision than others.

## 6. Conclusions

This paper addresses state-of-the-art investigations of the techniques proposed for the diagnosis of melanoma. In addition, there have been identified problems and difficulties. In addition, deep learning-based strategies such as convergent neural networks, pre-trained models, transfer learning and hybrid methods for detecting melanoma were studied in this research. It has been noted that deep learning techniques are essential for complex and composite preprocessing techniques such as picture resize and cropping, as well as pixel value norms. In addition, the critical limitations of existing approaches are described in this analysis as the areas where more changes are required. The hand-made techniques were better than the standard methods for deep learning. Many studies have employed designed features for the preprocessing and segmentation extraction capabilities. In addition, in medical image libraries, marking photographs is deemed the most critical task. A broad number of established benchmarks were made available to researchers to test their work, including PH2, ISBI, Derm IS, Dermquest, Med node, and Open Access datasets. In addition, melanoma diagnosis was also available in unpublished/non-listed data sets. However, it is hard to compare. For the classification of melanoma, various databases were available. Such datasets were freely accessible, and others were not available. It has been found that the numbers of images differed in multiple datasets. Moreover, some articles rendered a self-collected image dataset using the website. In the end, different challenges and future works are mentioned.

## Figures and Tables

**Table 1:** The research questions.

Research Question	Motivation
RQ1: What are the features and advantages of recently developed DL(Deep learning) methods for skin cancer classification?	This question helps to know about different techniques which have been proposed recently and their features
RQ2: What data sets are used in skin cancer detection methods evaluations?	This question helps to know about the publicly available data set that can be used in future research as a benchmark
RQ3: What are future challenges reported by papers in this domain?	This question helps to know about the future challenges in this domain
REQ:4 What are the machine learning and deep learning approaches for skin cancer detection	This question helps to know about the different methods used to detect skin cancer

**Table 2:** Primary and secondary keywords.

Primary Keywords	Secondary Keywords
Skin cancer, deep learning, machine learning skin cancer, detection and classification, lesion detection	Melanoma, carcinoma, lesion detection, medical image processing, system, framework algorithm

**Table 3:** The search query process.

Phase	Process	Selection Criteria	IEEE	Scopus	Willey	Google Scholar	Sprinkle	ACM (Association for Computing Machinery)	Science Direct	Total
1	Searching	Keywords	20	3	3	26	7	4	12	75
2	Searching	Title	20	3	2	20	7	4	7	63
4	Further Screening	Introduction and Conclusion	20	3	2	20	6	4	7	62
5	Evolution	Complete Articles	20	3	2	20	6	3	6	60

**Table 4:** Year-wise selection of papers.

Publication Year	No of Papers
2018	14
2019	14
2020	12
2021	10
2022	10

**Table 5:** Final paper selection.

IEEE	Scopus	Willey	Google Scholar	Sprinkle	ACM	Science Direct	Total
12	3	2	27	6	3	7	60

**Table 6:** The quality assessment of the papers.

Reference	Medium	Year	Quality Assessment				
			(a)	(b)	(c)	(d)	Score
[9]	A	2018	-	-	-	-	*
[10]	B	2013	-	-	-	-	*
[11]	B	2019	-	-	-	-	*
[12]	C	2020	-	-	-	-	*
[13]	C	2019	#	-	-	-	/
[14]	C	2018	-	-	-	-	*
[15]	C	2019	-	-	-	-	*
[16]	C	2007	-	-	-	-	*
[17]	C	2019	-	-	-	-	*
[18]	B	2013	-	-	-	-	*
[19]	C	2018	-	-	-	-	*
[20]	A	2020	#	-	-	-	/
[21]	C	2019	-	-	-	-	*
[22]	C	2018	-	#	-	-	/
[23]	C	2020	-	-	-	-	*
[24]	C	2020	-	-	-	-	*
[25]	C	2019	-	-	-	-	*
[26]	B	2020	#	-	-	-	/
[27]	C	2020	-	-	-	-	*
[28]	B	2019	1	1	-	-	4
[29]	C	2019	1	1	1	1	4
[30]	C	2019	0	1	1	1	3
[31]	C	2018	1	1	1	1	4
[32]	C	2018	1	1	1	1	4
[33]	C	2020	1	1	1	1	4
[34]	B	2019	0	1	1	1	3
[35]	B	2018	1	1	1	1	4
[36]	C	2018	1	0	1	1	3
[37]	C	2018	0	1	1	1	3
[38]	B	2019	1	1	1	1	4
[39]	C	2019	1	1	1	1	4
[40]	C	2018	1	1	1	1	4
[41]	C	2020	1	1	1	1	4
[42]	C	2020	1	1	1	1	4
[43]	C	2021	1	1	1	1	4
[44]	C	2021	1	1	1	1	4
[45]	C	2021	1	1	1	1	4
[46]	C	2021	1	1	1	1	4
[47]	C	2021	1	1	1	1	4
[48]	C	2022	1	1	1	1	4
[49]	C	2022	1	1	1	1	4
[50]	C	2022	1	1	1	1	4
[51]	C	2022	1	1	1	1	4
[52]	C	2022	1	1	1	1	4
[53]	C	2022	1	1	1	1	4
[54]	C	2022	1	1	1	1	4

In the quality assessment, part 0 indicates #, 1 indicates -, 3 indicates /, and 4 indicates *.

**Table 7:** Comparison of different DL methods.

Reference	Classification Techniques	Data Set	Performance Evaluation			
			Sensitivity of Method (%)	Specificity of the Method (%)	Precision (%)	Accuracy (%)
[19]	Transfer learning	PH2	98.4	98.8	97.7	98.7
[20]	Alex Net TL	PH2	100	96.7	Not given	97.5
[21]	DCNN	PH2	72	89	Not given	80.5
[22]	FRCN (Full resolution Conv. Network)	PH2	91.6	96.5	Not given	94.6
[23]	HRFB (High resolution Feature blocks)	PH2	96.44	94.2	Not given	94.9
[24]	3D CTF (Color Text features)	PH2	98.2	93.8	Not given	97.5
[25]	Depth-wise residual convolutional network	PH2	100	Not given	90.1	96.5
[26]	Transfer learning	PH2	92.5	94.5	Not given	93.3
[27]	DCNN (pixel-wise)	PH2	93.1	95.1	Not given	95.4
[28]	FCNN + Google Net	ISBI (2016) challenge data set	69.1	93.6	Not given	88.2
[29]	Transfer learning	ISBI (2016) challenge data set	90.2	99.1	92.1	92.5
[30]	Fusion Method (DCNN + Features)	ISBI (2016) challenge data set	93.2	80.5	Not given	95.6
[31]	OCF (Optimized color features) + DCNN	ISBI (2016) challenge data set	92.1	90.1	Not given	92.2
[32]	Fusion method (DCNN + Feature vectors)	ISBI (2016) challenge data set	Not given	Not given	68.9	86.9
[33]	FCN + Google Net	ISBI (2017) challenge data set	81.3	86.3	Not given	85.4
[34]	LDA + CNN	ISBI (2017) challenge data set	52.5	97.6	55.3	85.4
[35]	Transfer Learning	ISBI (2017) challenge data set	95.6	95.3	97.4	95.6
[36]	DCNN + Augmentation Algorithm	ISBI (2017) challenge data set	Not given	Not given	73.9	89.2
[37]	CNN + Ranking Algorithm + Ra Pooling	ISBI (2017) challenge data set	60	88.7	Not given	84.4
[38]	Transfer Learning Algorithm	ISBI (2018) challenge data set	80.2	98.1	Not given	97.6
[39]	CNN + Regularize	ISIC data set	94.3	93.2	Not given	97.6
[40]	ECOC + SVM + DCNN	Random images data set	97.0	90.2	Not given	94.3
[41]	Fusion method (Alexnet + VGG16)	Multiple	99.3	98.4	Not given	99%
[42]	Modified CNN	Multiple Dermis + Der Quest	94.2	94.5	Not given	94.6

**Table 8:** A list of publicly available datasets for skin cancer detection.

Reference	Dataset Name	Data Set Characteristics	
		Training Images	Testing Images
[9]	ISBI (2016)	273	900
[10]	ISBI (2017)	374	2000
[11]	ISBI (2018)	1113	10,000
[12]	ISBI (2019)	4522	25,333
[13]	PH2	40	200
[14]	ISIC	21,659	23,906
[15]	Dermot fit Images archive	76	1300
[17]	Dermis	146	397
[18]	MED-Node	100	170

**Table 9:** The future challenges reported in this domain.

Reference	Category	Discussed Future Challenge
[60]	Limited public data set	Since public data sets are not accessible, non-public databases and photographs gathered via the Internet are used for research. This complicates the replication of the findings since the dataset is not available.
[26]	Light-colored skin images	Since 2016, ISIC has arranged an annual melanoma diagnostic competition, but the presence of only light-skinned data is one of the drawbacks of ISIC. Dark-haired photographs are needed to be included in the datasets.
[21]	Lesion size impact on training	The lesion scale has also been found to be significant in most studies if the lesion scale is smaller than 6 mm, so melanoma cannot be identified, and the sensitivity of the diagnosis falls significantly.
[57]	Deep learning accuracy improvement	Deep learning approaches have been found to work correctly for 70% of training pictures and 30% of testing pictures. In comparison, findings require that the training ratio is necessary for good outcomes. The deep learning methods work well where the optimum balance is set. It is challenging to devise hybrid strategies that can work better with fewer training ratios.
[26]	Non-availability of fusion methods	Most of the techniques are focused on basic deep learning methods. However, fusion techniques are reported with better accuracy. Despite this, the fusion techniques are less reported in the literature for specific data sets.

**Table 10:** Machine learning and deep learning approaches.

References	Year	Approach	Result
[63]	2020	Two preprocessing methods and an automatic segmentation method based on semi-supervised learning are provided for usage with the offered dermoscopy images.	Deep learning techniques will be used in future research to improve the accuracy of the calculated coefficient values.
[64]	2018	This paper creates a novel approach for diagnosing lesions using deep learning and a localized feature encoding system.	Generate various feature values to work among a high amount of variation of lesions.
[39]	2019	In this article, we investigate the convolution neural network, a deep learning system that uses dermoscopy images to predict small skin changes.	The model’s performance is validated using images of lesions and the area under the curve for a lesion.
[65]	2019	Therefore, the manual analysis will have less room for interpretation and bias. Skin images may be automatically examined for melanoma and differentiated lesions using a deep learning method for working with lesion patterns.	Convolutional neural networks such as Inception-v4, ResNet-152 and DenseNet-161 were used to classify images of melanoma and skin discoloration plasma. To generate lesion segmentation masks, two U-Nets were trained.
[66]	2018	In the HAM10000 collection of dermoscopy images, skin cancer is recognized using the CNN model as an identifier.	RNN and other deep learning algorithms provided the most accurate cancer diagnoses, according to the results.
[67]	2019	An automatic system to improve the performance of classification for the efficient diagnosis of melanoma.	Results are reported for both segmented and non-segmented picture classifications. To expand the scope of the work, probabilistic graphical models can be added to this network.
[68]	2022	This paper offers a new judgement system known as an NN classifier that may more accurately diagnose skin lesions using deep learning methods such as neural networks and feature-based algorithms.	The stage of classification is implemented using SVM. The results obtained by the proposed system cover higher accuracy.
[69]	2019	The ABCD rule, GLCM and HOG algorithms are described as being used for feature extraction. Utilizing geodesic active shape, the lesion is separated to provide access to its features.	We categorized items with a sensitivity of 97.8% and a specificity of 0.94 using SVM classifiers. KNN’s application produced sensitivity and specificity of 86.2% and 85.0%, respectively.
[69]	2019	The majority of research is focused on separating melanoma-causing lesions from those that are not.	Our system of classifying skin tumors was shown to be accurate. Our results imply that doctors might be able to use the suggested technique to identify AM early.

## Data Availability

Not applicable.
